# Walter Butler Cheadle

**Published:** 1910-04-02

**Authors:** 


### OBITUARY.

The death is reported of Walter Butler Cheadle, M.A., M.D., F.R.C.P., in his 74th year. Dr. Cheadle, an old St. George's man, was on the consulting staff of St. Mary's and the Great Ormond Street Hospitals, and a late censor and examiner in medicine of the Royal College of Physicians, London, and examiner to the Universities of Durham and Cambridge. He was Lumleian lecturer in 1900, and Harveian lecturer, and the author of several books and monographs on medical subjects.

